# Knowledge and attitudes to personal genomics testing for complex diseases among Nigerians

**DOI:** 10.1186/1472-6939-15-34

**Published:** 2014-04-27

**Authors:** Lawrence Fagbemiro, Clement Adebamowo

**Affiliations:** 1 National Drug and Poison Information Centre, Federal Ministry of Health, Abuja, FCT, Nigeria; 2 West African Bioethics Training Program, University of Ibadan, Ibadan, Nigeria; 3 Institute of Human Virology, Abuja, FCT, Nigeria; 4 Department of Epidemiology and Public Health, Institute of Human Virology and Greenebaum Cancer Center, University of Maryland School of Medicine, Baltimore, MD 21201, USA

**Keywords:** Genomics testing, Personal, Ethics, Abortion, Religion, Complex diseases

## Abstract

**Background:**

The study examined the knowledge and attitudes to personal genomics testing for complex diseases among Nigerians and identified how the knowledge and attitudes vary with gender, age, religion, education and related factors.

**Methods:**

Data were collected using qualitative method in 2 districts of the Federal Capital Territory. In the study, eight (8) Focused Group Discussions (FGDs) and twenty seven (27) Key Informant Interviews (KIIs) were conducted. Participants for the research were recruited among healthy Nigerians, individuals with complex diseases, health care professionals, community leaders and health policy makers.

**Result:**

Analysis of the result showed that most respondents in both FGDs and KIIs had limited knowledge about genomics test initially. Their understanding of the test however improved after explanation on its concept. Participants showed positive attitude towards genomics tests. Nevertheless they expressed fear over direct to consumer personal genomics testing, testing unborn babies and disclosure of results to third parties. Culture and religion were found to influence the perspectives of respondents on genomics test particularly those aspects that could either directly contradict their beliefs and practices or lead to actions which contradict them.

**Conclusion:**

In conclusion, most Nigerians interviewed had limited knowledge of genomics test but with supportive attitude towards its use in predicting future risk of complex diseases after understanding the test concept. Genomics testing for complex diseases was not a common practice in Nigeria.

## Background

Advances in medical technologies have made genomics tests that predict risk of diseases increasingly available for use in clinical settings. With the substantial reduction in cost and its rapid spread around the world, it is inevitable that genomics tests for complex diseases will soon be available everywhere including Africa. As the number of these tests increases, the uses and interpretation of the information they generate will require increased understanding of genomics and how its principles apply to different health problems. Such uses are raising concerns about the ethical issues that may arise when these technologies are used to identify genetic markers of disease risk in otherwise healthy people. The concerns including risk of discrimination by schools, insurers, or employers [1] are pertinent given that hundreds of genetic markers associated with a variety of complex diseases, including cancer, diabetes, cardiovascular disease, and Alzheimer disease have been identified and some of these are now in clinical use [2].

Increasing availability of genomics tests for non-communicable diseases (NCD) in Nigeria and other African countries raises the same types of questions as in developed world. However, there may be additional ethical dimensions because of the peculiarities of Africans.

Majority of Africans are poor and have high levels of illiteracy [3], the level of comprehension of genomics and genomics risk of diseases is therefore uncertain. While the concept of heritability is well known, subtle differences such as those between Mendelian or multi-gene risk of disease may be more difficult.

Africans have specific beliefs about origin of illnesses and health [4], and this may affect their willingness to do these tests, believe the results and act on the results.

Africans have specific stories of origin, kinship and personhood that may be challenged by the result of genomics tests and the impact of such interventions on sense of identity is unknown at this time.

Africans may not have ready access to interventions that will change the outcomes if they know their genetic risk of certain diseases. This raises questions about the ethical implications of conducting such tests and whether the researchers/test laboratories have an obligation to provide the required interventions.

To this end, we conducted a qualitative study in Nigeria, which is the most populous African countries to assess their attitude to genomics tests for complex diseases, using Focused Group Discussion (FGD) and Key Informant Interviews (KII). Though Nigeria is the most populous country in Africa, similar studies need to be conducted in more African countries to get the general view of Africans.

## Methods

A cross-sectional qualitative study was carried out in 2 districts (1 rural and 1 urban) of Abuja, the Federal Capital Territory (FCT) in Central Nigeria. We conducted 8 FGDs and 27 KII using topic guides and prompt statements to elicit participants’ knowledge about general genomics issues, their attitude to personal genomics testing for complex diseases and its determinants. The KII was to extract more in-depth responses after the FGDs.

Methodological and data triangulation were used to gain different perspectives on personal genome testing and to establish the validity of the research studies by comparing divergence and similarities between the findings from the different data sources and methods [5].

The study was conducted from January to March 2011 and participants were purposively selected.

### Research setting

Abuja’s population was estimated at 778,567 in 2006 with growth rate of up to 30% a year [6]. Due to its role as the capital of Nigeria, individuals from all ethnic groups, tribes and religions in Nigeria live there. Muslims make up approximately 50% of the population, Christians 40%, while the remaining people adhere to indigenous beliefs^4^. Abuja has five districts and several surrounding towns and villages. We selected Asokoro (Urban) and Bwari (Rural) districts for this study. We chose a rural and an urban setting for this study in order to ensure adequate representation of all participants from diversity of socio-economic background.

### Data collection

#### Focus Group Discussions (FGDs)

We conducted 4 FGDs in the urban and 4 in the rural districts. The categories of participants in each FGD were: adult male, adult female, youth male and youth female (youths were selected from 18 yrs to less than 25 years). We ensured that participants in each focus group were balanced according to the following criteria – Level of education, Age, Sex, Tribe and Religion. Letters were sent to participants detailing the objective of the study and inviting them to participate in the study. Each session had 10 participants and was conducted by a moderator and a recorder. Participants’ categorization was based on age and gender because of paternalism that are prevalent in some Nigeria cultures which might prevent women and young persons from freely expressing their opinions in a mixed group. FGDs were conducted separately for men and women also because of religious sensitivities in Nigeria. Knowledge of participants was assessed using open ended questions and they were thereafter educated on the concept before assessing their feelings about the test.

We conducted a pilot FGD at Karu district of Abuja to test the study instruments for comprehensibility and usability. We used the results of the pilot study to modify our instruments accordingly.

#### Key Informant Interviews (KII)

We conducted KII with selected health workers who provide care for individuals with complex diseases, individuals with complex diseases, relatives of people with complex diseases, religious leaders, community leaders and opinion leaders. Respondents were purposively selected to ensure adequate representation by individuals who can give adequate and relevant information. Tables 1, 2 and 3 show the distribution of participants in the KII.

**Table 1:** Matrix of Key Informant Interview Participants’ Selection among the General Population in the 2 Districts

**Category**	**No of Participants per district**		**Total**
	**Urban**	**Rural**	
Community leader	1	1	2
Opinion leader (male)	1	1	2
Opinion leader (Female)	1	1	2
Christian religious leader	1	1	2
Muslim religious leader	1	1	2
Traditionalist	1	1	2
Head teacher	1	1	2
Student	1	1	2
Total	8	8	16

**Table 2:** Matrix of Key Informant Interview Participants’ Selection at Health Institutions in Urban Districts

**Category**	**No of Participants**
Hospital Administrator/MD	1
Med Lab Scientist	1
Medical Doctor	1
Nurse	1
Pharmacist	1
Patient with a complex disease	1
Relative of patient with a complex disease	1
Total	7

**Table 3:** Key Informant Interview Participants’ Selection among Policy Makers at Federal Ministry of Health, Abuja

**Category**	**Total No of Participants**	**Remark**
National Health Policy Maker	3	Director Health Planning and Research, Director Hospital Services and Deputy Director, laboratory services at Federal Ministry of Health(FMOH)
Ethicist	1	FMOH
Total	4	

The KII were conducted using semi-structured discussion guide. The guides were scenario-based and were updated with information derived from the FGDs in order to elicit more in-depth responses by using follow-up questions, prompts and probes. The interviews lasted an average of 30 minutes, were audio-taped, transcribed and verified by the interviewer prior to analysis.

### Data analysis

The interviews were transcribed. In cases where interviews were conducted in local languages, they were translated to English language and back translated into the local languages to check consistency and accuracy. Information received was presented verbatim, preserving language and concept used. Data was analysed using open coding and grounded theory to ensure that all the different issues, perceptions and practices that arose from the interview transcripts were coded. The first analysis was done by two researchers; the coding and sub-coding were done manually to show linkages and networks between the different themes. An independent researcher reviewed the coding and themes, to ensure that they reflected the different issues that arose from the interviews. Category headings were generated from the data and all of the data were accounted for under these. Two independent researchers verified the accuracy of the categorization and minor modifications were made to it after their input.

Ethical approval for this study was obtained from the National Health Research Ethics Committee (NHREC) of Nigeria.

## Results

### Focus group discussions

A total of 80 individuals participated in the 8 FGDs. Their ages ranged from 18 to 68 years with mean of 39.5 and SD 20.2 years. There were 39 women (48.75%) and 41 men (51.25%); 20 Yorubas (25%), 20 Ibos (25%), 21 Hausas (26.25%) while 19 participants (23.75%) were from other minority ethnic groups. There were 38 Muslims (47.5%) and 42 Christians (52.5%). There were 30 students (37.5%), 20 civil servants (25%), 15 traders (18.75%), 5 teachers (6.25%), 2 health care professionals (2.5%), 2 clergies (2.5%) and 6 from other professions (7.5%).

A. **Knowledge of Genomics Tests**

From the FGD, most respondents’ knowledge of genomics tests was limited to paternity and sickle cell genotype tests during initial assessment. For example, a participant said:

“..genomics test is done when a couple has a baby and there is a concern about why the baby does not look like the father....”

Their understanding however improved after we educated them on the concept of genomics test. It was also noted that none of the participants ever had personal experience of genomics test.

B. **Regulation of Genomics Tests**

The majority of participants in the FGD saw genomics test as a professional issue that should be done within health facilities. Respondents believed that those in need of the service can be properly counselled before and after the test if done at health facilities. To quote a participant,

“…without a professional involvement, it is not ideal to run such tests. There may be errors in interpreting the result.”

Participants believed that genomics test results could be misinterpreted if not done by professionals. In addition, they felt that patients should have access to pre and post testing counselling.

On the contrary, some female youths in the rural and urban centres were of the opinion that genomics tests should be made available directly to the consumers with proper education on how to do them. They said, that would eliminate time wasted at health facilities and reduce the cost of doing such tests while increasing accessibility. One of the girls said,

“....it would be fine to do the test yourself if trained and know if you have future risk of getting a disease and if assistance is needed, the user can seek help of a health professional. ....... So people would not go to the hospital and waste precious time just to see a doctor for the test.”

Another female respondent, a student said the test should be made available directly to the consumer for confidentiality sake

“....if the test is done in the hospital or laboratory they may leak the result to a third party who may stigmatise you because of your genetic predisposition......this is not good …”

C. **Perceptions about relevance of Genomics Tests in Nigeria**

Most participants in the FGD believed that genomics tests were relevant in Nigeria, even if there was no access to intervention that would change outcome of the diseases whose risks could be detected with genomics test. Respondents said, doing the test could inform life style modification and family decisions by the tested individuals. A respondent said:

“It is still relevant to carry out genomics tests even though the necessary intervention may not be available in Nigeria. It is better to know than to be ignorant....... Knowledge they say is power. There are steps that can be taken which may not necessarily be medicine use. It may include lifestyle modification.”

D. **Willingness to do Genomics Tests**

All respondents in the FGD were willing to do genomics tests if available and affordable. Some male youth participants in the urban area in a chorus answer said:

“..if it is not expensive, we are willing to do genomics test....”

E. **Disclosure of Result of Genomics Tests to Third Parties.**

Generally, respondents in FGD frowned at disclosure of test result to a third party. However some young participants said if their consents were sought, the result can be disclosed to partners. In the same discussion, a few elderly respondents said they would be willing to disclose such result to their life insurance companies, one of whom said:

“....these are the people that will take care of me if I am sick, I think they should know.”

Participants expressed fear about disclosure of test result to employers because of the risk of losing their jobs.

F. **Testing and Disclosure of Result to Children.**

All participants agreed that children could be tested to predict if they would have serious diseases in future but the result should not be disclosed to them until age of maturity. It was stated that children might not be able to comprehend or handle such information. In addition, unfavourable result could cause irreparable emotional injury to children as some of them might see it as the end of life.

One respondent, a student said,

“Yes children should be tested. They should not know the results as they are not yet capable of taking care of themselves….only the parents should know.”

G. **Genomics Testing of Unborn Babies**

Majority of the respondents in the FGD did not see any reason why unborn babies should be tested because undesirable result would lead to dilemma of what to do next. One participant said:

“.... but when the result comes out what will you do? Will you abort the pregnancy? The best thing is to leave the unborn alone as testing will bring problems on what to do next when the result is not desirable.”

However some of the participants (4 women) in Asokoro district (urban) were of the opinion that unborn babies should be tested so that the parents could start taking precautions or abort the pregnancy if necessary.

One of the women said:

“It is good to test the unborn children. The earlier one knows the risk of diseases they are carrying, the better.”

H. **Effect of Religion and Culture**

Most respondents in the FGDs claimed that their attitudes to genomics testing were influenced by their religion or culture particularly as regards testing unborn babies. That was observed among respondents across different ethnic groups and religions. One of the women said:

“Not good to test the unborn because if the result is not favourable, it will result in abortion which is against my religion.”

### Key informant interviews

There were 27 participants in the KII. Their ages ranged from 21 to 67 years with mean of 42.0 and SD 13.6 years. There were 11 women (40.7%) and 16 men (59.26%), 8 Hausas (29.6%), 7 Ibos (26%), 7 Yorubas (26%) and 5 from other ethnic groups (18.4%). There were 16 Christians (59.26%) and 11 Muslims (40.74%). Nine (9) participants were into health care work (33.3%), 4 into education related activities (14.8%), 2 into business/commercial activities (7.4%) and 12 were community/religious leaders (44.4%).

A. **Knowledge of Genomics Tests**

Similar to the result obtained in FGD, most participants in KII showed limited knowledge of genomics testing except the young health care workers. That category of key informants had better knowledge of genomics tests than others. It was further observed that most of the key informants had no personal experience of genomics test.

B. **Regulation of Genomics Tests**

Majority of the respondents in the KII said gemonic tests should be conducted by professionals at designated centres because the concept was new in Nigeria.

C. **Perceptions about relevance of Genomics Tests in Nigeria**

Like participants in the FGD, all respondents in the KII said genomics tests would be relevant in Nigeria even with the lack of access to intervention that could change outcome of genetic risk at the moment. Health workers could help individuals with undesirable results think of what to do in order to alleviate the identified problem or prevent future manifestation. Results might also help in the formulation of future health plans for Nigerians by policy makers. A respondent said:

“It is relevant to still carry out the test even if interventions are not immediately available in Nigeria. It is better to know than not knowing because a pre-disposed individual could take some personal actions that may prevent the manifestation of the disease.”

D. **Willingness to do Genomics Tests**

All KII participants showed willingness to do genomics tests if and when it becomes widely available in Nigeria. One interviewee said:

“....I am willing to do genomics tests if available as it will help one to know if there is any future problem that can quickly be aborted.”

E. **Disclosure of Result of Genomics Tests to Third Parties.**

Most respondents in the KII said they would not want their test results disclosed to anyone except their health insurance company and their spouses. A male opinion leader in a contrary view said the test result could be disclosed to anyone or organisation that could be affected by the result of the test. Another respondent in the KII however vehemently said the test result should not be disclosed to a third party no matter the condition. He said:

“It is a confidential test....I will not want the result of the test to be disclosed to any third party under any condition.”

F. **Testing and Disclosure of Result to Children.**

The KII interviewees also had no hesitation about testing children but the result should be kept away from them until they are matured.

G. **Genomics Testing of Unborn Babies**

Most interviewees expressed displeasure at the testing of unborn babies. A medical doctor added that the process of testing might also lead to abortions; hence he was not in support of testing unborn babies. A few respondents said testing unborn babies early in pregnancy would help the mother decide if termination of the pregnancy was necessary. A respondent said:

“…if test is done early enough, the pregnancy can be terminated if that will prevent future agony.”

H. **Effect of Religion and Culture**

In the KII, majority of the respondents said their perceptions of genomics test were affected by religion in situations where the result of test could lead to abortion or killing of individuals with undesirable results. One said:

“....my religion will not allow me to support anything that takes life, so if genomics test will lead to abortion or killing, I am not in support.”

## Discussion

This study showed that most of the respondents, except a few young health workers did not have a good knowledge of genomics test prior to educating them on the concept. It was notable that the participants showed positive attitudes towards genomics tests and potential benefits despite that most of them lacked personal experience of the tests. A significant proportion expressed worry over personal genomics testing, testing of unborn babies and disclosure of test results to a third party. Some participants also confused personal genomics test to detect risk of complex diseases with sickle cell genotype or paternity tests.

A study by Pagan *et al.* on possible causes of low awareness of genetic test information among African Americans showed that educational level and place of residency were relevant contributing factors [7]. In our study, we did not find any difference between the awareness of participants who resided in urban areas and the outskirts. Conversely, the level of awareness of young health workers interviewed was relatively higher than that of others, probably on account of their modern medical training and clinical experience on genomics tests.

### Regulation of genomics test

In the US, geneticists, general public, consumer advocates, and government bodies have raised alarm about Direct to Consumer Genomics Tests and the risk that consumers could be misled by false or misleading claims leading them to make harmful healthcare decisions on the basis of test results [8]. In our study, most respondents expressed concerns about misinterpretation of test results if it was not handled by well trained professionals. On the contrary, younger female respondents supported out-of-hospital genomics tests because they believed these would be more confidential and the time wasting usually associated with hospitals/laboratories visits would be eliminated. The view of the young women might be due to their experience of health care systems which they interact with more than men on account of pregnancies. Their attitude may also be because of greater familiarity with home based tests such as pregnancy tests and they have come to appreciate the ease and confidentiality involved. Similarly, Kolor *et al.* in 2009 in their study reported that women had a more positive attitude towards personal genomics tests than men [9].

### Relevance of genomics test in Nigeria

In this study, respondents generally believed that conducting genomics tests was relevant even if there was no access to intervention that would change the outcome. They emphasised that the test result could help in life style modification, family decision and formulation of future health plans for Nigerians by policy makers. This is similar to the findings of Walker in 2007 on Huntington’s Disease where he reported that some respondents chose testing for Huntington’s Disease despite that there is no treatment for the condition but as an aid in career and family decisions” [10].

### Willingness to do genomics tests

In this study, respondents expressed willingness to do genomics tests to predict future risk of a complex disease if offered the opportunity to do so. Their willingness was independent of age, literacy level or sex. This contrasts with the result of study on primary care patient by Masanobu *et al.* in 2013 where age, level of education, family history of disease positively correlated with willingness to undergo genomics testing for salt-sensitive hypertension, and other studies on willingness to be tested for genetic risk of cancer and other hereditary disease [11].

### Disclosure of result to third parties

This study revealed that many people were sceptical about disclosure of test results to third parties. Most respondents who agreed to disclose test results would only do so to their spouses and health insurance companies. That decision might be because of the role health insurance companies and spouses play during sickness. Health insurance as a means of providing funding for health care is a novelty in Nigeria and there was no particularly discernible attitude to them and their work in Nigeria at present. That might change in future. None of the study participants agreed to disclose genomics tests results to employers because of the risk of losing jobs. The result was similar to the finding of Amy Harmon in 2008 that some individuals avoided genetic testing out of fear that it would affect their ability to find a job or keep an existing one if undesirable results were disclosed to employers [12]. There was only one discordant voice in the KII who said the test result could be disclosed to anybody that might be affected by the outcome of the result even without his consent. That discordant voice was in line with the view of the majority of foreign jurisdictions, which were in favour of limited disclosure of genetic test results (without the consent of the patient) in cases where the harm to “at-risk” relatives was grave and imminent and the information could result in effective intervention [13] ( Knoppers *et al.* 1998).

### Testing and disclosure of result to children

Similar to the result obtained by Keneth *et al. in* 2011, as quoted “parents viewed the benefits of pediatric testing to outweigh its risks (positive decisional balance) and were interested in pediatric testing,” [14] respondents in the study were of the opinion that children could undergo genomics tests but the result of such tests should not be disclosed to the children until maturity. The reason most commonly cited for non-disclosure of result to minors was because they were too young to comprehend and take decisions on the basis of the result. Many parents thought their children might worry about a positive result, making them unlikely to enrol their children, or to choose not to tell the child test results [15] (Bernhardt et al. 2003). Many ethicists believed that genetic testing for adult-onset conditions generally should be deferred until adulthood or until an adolescent interested in testing has developed mature decision-making capacities [16] (Committee on Bioethics.2001).

### Testing unborn babies

Most participants were against testing unborn babies because of the risk of harming the foetus and the dilemma of what to do next if the result was unfavourable. They did not want to be put in a position where they would have to consider abortion of the pregnancy as that would tempt their religious beliefs/faith. Similarly, research by Adeola *et al.* in 2012 on prenatal screening for sickle cell anaemia showed that many respondents would not allow preventive abortion of pregnancy if screening was positive because of their religion [17]. “Termination of pregnancy by abortion even when there is future risk to the foetus is considered a sin particularly among Christians” [18] (Kagu et al. 2004).

### Effect of religion and culture

Our respondents claimed that religion and culture affected their attitude to genomics testing particularly those aspects that might either directly contradict their beliefs and practices or lead to actions that contradicted their religious beliefs. Similarly, a study conducted by Thomas in 2007, showed that there was a strong religious influence on attitudes and approaches towards genomics test testing [19].

## Conclusion

From the study, we concluded that most Nigerians interviewed had limited knowledge of genomics test. However, they showed supportive attitude towards its use in predicting future risk of complex diseases after understanding the test concept. They frowned at testing of unborn babies, unregulated availability of test, disclosure of test results to children and a third party. Culture and religion affected the views of respondents on genomics test. Genomics testing for complex diseases was not a common practice in Nigeria.

### Limitations of the study

Our study was limited by the use of only qualitative methods but the findings lay a foundation for more research using other methods to further probe the responses obtained. In addition, the study was restricted to Abuja, Nigeria’s capital. Even though Abuja is a cosmopolitan city with different Nigerian tribes, yet conducting the study in different parts of the country may reveal different results. Also, the study was limited by the number of participants, which might not have been a true representation of the beliefs and attitudes within the entire Nigerian population. However it is hoped that the attempt to recruit participants from different ethnicities and religions, gender and age, would allow a fair representation of different ethnic and religious beliefs.

The study did not explore the willingness of Nigerians to have genomics tests for specific diseases like Alzheimer and Huntington Diseases where positive result may carry severe consequences. Some respondents might not have fully understood the concept of genomics test despite the background information given during the interviews, because genomics test is not a common practice in Nigeria.

Giving the participants a number of multiple choices or true/false test questions might be a better method to assess knowledge on genomics test but we used open ended questions for that part of the study.

## Competing interests

The authors declare no conflict of interest.

## Authors’ contributions

FL conceived the research, carried out sample selection, participants’ interview and drafted the manuscript. CA vetted the research design, result and manuscript. He also helped in securing grant for the research work. Both authors read and approved the final manuscript.

## Authors’ information

The work titled Knowledge and Attitude to Personal Genomics Testing for Complex Diseases among Nigerians was done by FL (principal and corresponding author) and CA (co-author).

FL is a clinical pharmacist in the Federal Ministry of Health. He is the Head of National Drug and Poison Information Centre, Nigeria.

Lawrence attended University of Ibadan where he graduated with a B.Pharm degree. He obtained M.Pharm degree(Clinical Pharmacy) from University of Lagos and was also trained in biomedical science in University College of Medical Science, New Delhi, India.

Until now, he was into clinical pharmacy and saddled with the responsibility of ensuring regular review of National Essential Medicine List and National Standard Treatment Guidelines for Nigeria.

In the last few years, he has shown interest in Bioethics debates and research. His newly developed love for bioethics made him to proceed to University of Ibadan for M.Sc Bioethics degree through the West African Bioethics Programme. Lawrence currently represents his Department in all bioethics issues within and outside Federal Ministry of Health. His research interest includes bioethics, genomics of complex diseases, toxicogenomics and pharmacogenomics.

Dr. Clement Adebamowo is Professor of Epidemiology and Public Health at the University of Maryland, Baltimore, MD 21201; Director of Research, Institute of Human Virology, Abuja, FCT, Nigeria and formerly Professor of Surgery at the University of Ibadan. He is Program Director of the West African Bioethics Training Program, Nigeria.

Both authors worked together throughout the period of this research.

## Pre-publication history

The pre-publication history for this paper can be accessed here:

http://www.biomedcentral.com/1472-6939/15/34/prepub
